# Perioperative neurocognitive disorders: Advances in molecular mechanisms and bioactive molecules

**DOI:** 10.1016/j.arr.2025.102885

**Published:** 2025-09-04

**Authors:** Liwei Mao, Lian Wang, Zhihai Huang, Jeffrey A. Switzer, David C. Hess, Quanguang Zhang

**Affiliations:** a Department of Neurology, Medical College of Georgia, Augusta University, 1120 15th Street, Augusta, GA 30912, USA; b Department of Neurology, Louisiana State University Health Sciences Center, 1501 Kings Highway, Shreveport, LA 71103, USA; c Institute for Cerebrovascular and Neuroregeneration Research (ICNR), Department of Neurology, LSU Health Shreveport, 1501 Kings Hwy, Shreveport, LA 71103, USA

**Keywords:** Perioperative neurocognitive disorders, Neuroinflammation, Epigenetic modifications, Noncoding RNA, Gut–brain axis, Mitochondrial function

## Abstract

Perioperative neurocognitive disorders (PNDs) are common complications following surgery, especially in elderly patients, and are characterized by memory loss, attention deficits, and impaired executive function. The pathogenesis of PNDs involves a complex interplay of neuroinflammation, neurotransmitter imbalance, epigenetic modifications, and gut–brain axis disruption. This review summarizes the latest findings on the mechanisms underlying PNDs, with a focus on microglial activation, interleukin imbalance, and NLRP3 inflammasome-mediated pyroptosis. We further discuss how anesthesia and surgery impair synaptic plasticity by disrupting neurotransmitter systems, particularly GABA_A_ and NMDA receptors. Additionally, we highlight the roles of epigenetic regulation and gut microbiota dysbiosis in sustaining neuroinflammation and cognitive deficits. Finally, we explore potential therapeutic strategies, including bioactive compounds and neuroprotective agents, offering new directions for the prevention and treatment of PNDs.

## 1. Introduction

Perioperative neurocognitive disorders (PNDs) comprise a broad range of cognitive and behavioral disturbances associated with the perioperative period, notably including preoperative cognitive impairment, postoperative neurocognitive disorder, postoperative delirium (POD), and delayed neurocognitive recovery (DNR), which are particularly prevalent in elderly surgical patients (Evered et al., 2018). POD is a common and serious complication that typically occurs within the first 7 days after surgery. Its incidence ranges from 13.2 % to 44 %, depending on surgical type and patient condition, and is associated with a 30-day mortality rate of 7–10 % (Jin et al., 2020; Ansaloni et al., 2010; Robinson et al., 2009). A meta-analysis of patients undergoing noncardiac surgery reported a 18 % incidence of POD, which was significantly associated with advanced age, low body mass index, impaired preoperative cognitive function, and elevated inflammatory markers (Sadeghirad et al., 2023). DNR typically refers to cognitive dysfunction occurring within 30 days after surgery. Among patients undergoing non-neurological and non-cardiac procedures, the incidence of DNR ranges from 25.8 % to 34.5 % within the first postoperative week, declining to 7–11 % by three months (Moller et al., 1998; Monk et al., 2008; Paredes et al., 2016). With the aging population, anesthesia- and surgery-related neurocognitive disorders have emerged as a pressing clinical concern.

Optimizing anesthesia techniques can reduce various postoperative complications. Regarding anesthetic selection, propofol-based anesthesia has been shown to lower the risk of postoperative cognitive dysfunction in cancer patients compared to sevoflurane, although no significant difference was observed in laparoscopic procedures (Zhang et al., 2018a; Wang et al., 2019; Li et al., 2021a). Moreover, combining propofol with esketamine has been reported to enhance postoperative cognitive recovery in elderly surgical patients (Tu et al., 2021). Opioid analgesics and sedatives facilitate postoperative cognitive recovery, as uncontrolled pain is a major contributor to PNDs, especially in elderly patients (Wang et al., 2007; Awada et al., 2019). However, clinical studies often compare these interventions to placebo, and prolonged use of high-dose analgesics and sedatives has been linked to central nervous system damage, further underscoring the association between surgical procedures and cognitive impairment (Khani et al., 2022; Wouters et al., 2020). Therefore, PNDs and their recovery involve complex processes influenced by surgical type, anesthesia modality, and preoperative patient status. Elucidating the underlying molecular mechanisms and identifying key therapeutic targets are essential for developing effective prevention and treatment strategies.

Animal studies have shown that anesthesia, surgery, or traumatic brain injury significantly increases the risk of neurocognitive dysfunction, with effects extending to male offspring (Ju et al., 2023). The whole transcriptome sequencing of the hippocampus in aged mice across perioperative stages revealed neuroinflammation as the most prominently activated pathway. In addition, surgery-induced alterations in neurotransmitter and receptor function, neuronal and synaptic integrity, metabolic homeostasis, oxidative stress, circadian rhythms, and blood–brain barrier (BBB) integrity also contribute significantly to the development of PNDs (Suo et al., 2022; Xiang et al., 2019).

Despite growing insights into the mechanisms of PNDs, a unified understanding of their multifactorial nature remains lacking, and effective clinical strategies are still limited. Therefore, this review aims to comprehensively synthesize recent findings on the molecular and cellular mechanisms underlying PNDs, including neuroinflammation, neurotransmitter dysregulation, epigenetic alterations, and gut–brain axis disturbances, and to evaluate emerging therapeutic strategies targeting these pathways. By integrating mechanistic advances with translational potential, we seek to provide a scientific foundation for developing more effective preventive and therapeutic approaches for PNDs in clinical practice.

## 2. Neuroinflammation as a key contributor to the pathogenesis of PNDs

### 2.1. Microglia-mediated neuroinflammation

Microglia, the resident immune cells of the CNS, play a pivotal role in maintaining neural homeostasis through tightly regulated activation (Wyss-Coray, 2016). These highly dynamic cells exhibit remarkable morphological plasticity, shifting between a surveillant ‘resting’ state and an activated phenotype; in their resting state, microglia continuously scan the microenvironment via fine processes, facilitating synaptic pruning, remodeling, and neural circuit regulation (Nimmerjahn et al., 2005; Fetler and Amigorena, 2005). Upon sensing damage, microglia rapidly transition from surveillance to a reactive state, mounting a swift chemotactic response to protect injured tissue (Raivich, 2005). Using the radiotracer [^1 1^C]PBR28 to target microglial activation markers, positron emission tomography reveals robust CNS microglial activation following LPS-induced systemic inflammation in healthy humans (Sandiego et al., 2015). In some male patients undergoing prostatectomy under general anesthesia, microglia exhibited aberrant dormancy at 3–4 days post-surgery, followed by heightened activation at 3 months (Forsberg et al., 2017). Both immune dormancy and sustained microglial overactivation impair postoperative neurocognitive recovery.

In a sevoflurane-induced (a widely used inhalational anesthetic) PND mouse model, hippocampal expression of the triggering receptor expressed on myeloid cells-1 (TREM1) was markedly upregulated, driving microglial pro-inflammatory polarization and aggravating postoperative neuroinflammation–associated cognitive deficits (Tang et al., 2023a). Lipocalin-2 (LCN2) is an innate immune protein involved in regulating iron homeostasis and inflammatory responses under both physiological and pathological conditions (Xiao et al., 2017). Following tibial fracture surgery, LCN2 expression was markedly upregulated in hippocampal neurons (Xiang et al., 2022). Although LCN2 upregulation occurred independently of postoperative microglial activation, it promoted microglial pro-inflammatory polarization. Silencing neuronal LCN2 attenuated microglial activation and alleviated postoperative cognitive impairment. These findings highlight potential targets for modulating microglial activation and neuroinflammation after surgery.

In the sevoflurane-induced PND model, microglial hyperactivation was associated with downregulation of hyperpolarization-activated cyclic nucleotide-gated (HCN) channels—recently identified ion channels expressed on microglia (Xu et al., 2022; Vay et al., 2020). HCN channel blockade promoted pro-inflammatory microglial polarization, worsening cognitive deficits and anxiety-like behaviors. Conversely, surgery-induced microglial activation was shown to depend on voltage-gated Kv1.3 potassium channels, whose genetic deletion or pharmacological inhibition mitigated fracture-induced neuroinflammation and cognitive impairment in mice (Lai et al., 2020). These findings underscore the critical role of microglial ion channel states as key modulators of neuroinflammation.

In an aged mouse model of carotid artery exposure, surgical trauma elevated hippocampal glucose metabolism and shifted energy production from oxidative phosphorylation toward glycolysis (Luo et al., 2021a). Treatment with the glycolysis inhibitor 2-DG attenuated surgery-induced metabolic reprogramming, suppressed hippocampal microglial M1 polarization, and improved postoperative cognitive recovery. Additionally, another study reported reduced hippocampal expression of glucose transporter 1 (GLUT1) seven days after abdominal surgery in aged mice, leading to energy deficits, blood–brain barrier disruption, and cognitive impairment (Chen et al., 2023a). Whether impaired glucose metabolism directly influences microglial activation and neuroinflammation remains unclear. However, earlier evidence showed that abdominal surgery in aged rats disrupted hippocampal insulin signaling, triggering microglial activation and exacerbating neuroinflammation (Kawano et al., 2016). Surgery increased hippocampal glycogen synthase kinase 3β (GSK-3β) levels while suppressing its inhibitory phosphorylation (pGSK-3β^ser9^). Given the central role of insulin signaling in regulating glucose metabolism, further investigation is needed to determine whether its disruption contributes to metabolic dysfunction and exacerbates postoperative neuroinflammation.

In a PND mouse model induced by etomidate (a short-acting intravenous anesthetic), hippocampal analyses revealed marked microglial pro-inflammatory polarization one week post-surgery and subsequent activation of neurotoxic A1 astrocytes by week three. Notably, preoperative microglial depletion prevented A1 astrocyte activation and hippocampal cognitive impairment (Li et al., 2020). In a myocardial ischemia–reperfusion mouse model, activation of A1 astrocytes triggered postoperative delirium-like behavior in aged mice. This effect was primarily driven by hippocampal network imbalance caused by elevated extracellular glutamate. Excess glutamate suppressed pyramidal neuron activity and enhanced inhibition from parvalbumin-expressing interneurons (GABAergic inhibitory neurons) through tonic NMDA receptor activation (Liu et al., 2024a).

**Fig. 1.**
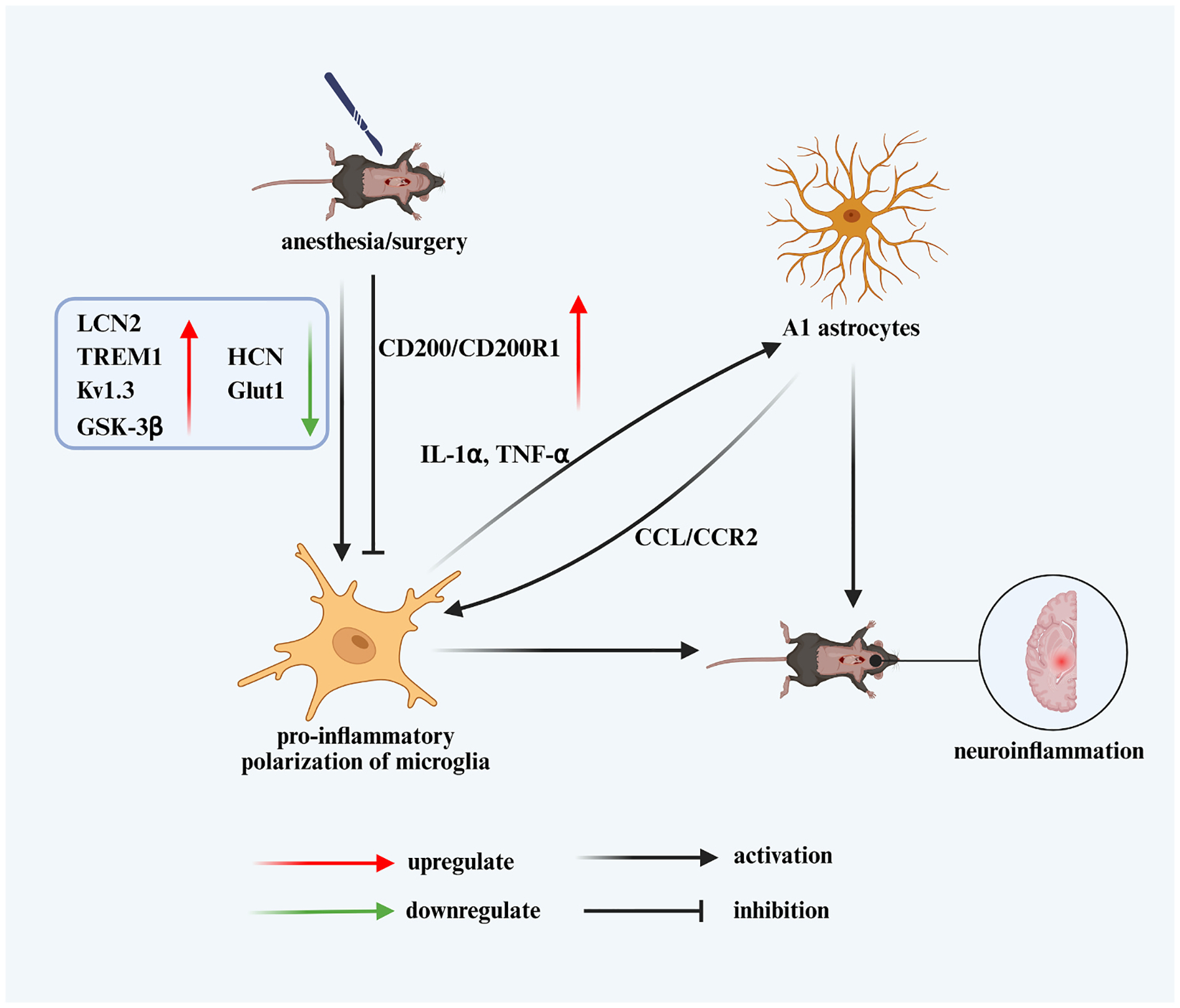
Microglia–astrocyte–neuron crosstalk mediates postoperative neuroinflammation. anesthesia and surgery trigger multiple molecular alterations in the hippocampus, including upregulation of LCN2, TREM1, voltage-gated potassium channel Kv1.3, GSK-3β, and neuronal membrane protein CD200/CD200R1 signaling, along with downregulation of HCN channels and GLUT1. These changes promote pro-inflammatory polarization of microglia through ion channel modulation, metabolic reprogramming, and impaired glucose metabolism, accompanied by increased production of IL-1α and TNF-α. Activated microglia induce neurotoxic A1 astrocyte transformation, while astrocyte-derived chemokine CCL2 stimulates microglial activation via CCR2 signaling, amplifying neuroinflammatory responses. CD200/CD200R1 interaction also regulates microglial activation status. The sustained feed-forward loop between microglia and A1 astrocytes culminates in post-operative neuroinflammation and cognitive dysfunction.

Following fracture surgery, hippocampal levels of astrocyte-derived chemokine C single bond C motif ligand 2 (CCL2) were elevated, promoting microglial activation and upregulation of chemokine receptor type 2 (CCR2) (Xu et al., 2017a). The interaction between the neuronal membrane protein CD200 and its receptor CD200R1 (mainly expressed in microglia) was also involved in the proinflammatory polarization of microglia after surgery (Ma et al., 2021). In a liver surgery mouse model, CD200 expression transiently increased on postoperative day 1 and normalized within 3–7 days, while treatment with a CD200R1 agonist markedly reduced neuroinflammation and improved cognitive function. These findings underscore the pivotal role of microglia–astrocyte–neuron crosstalk in orchestrating postoperative neuroinflammation and cognitive outcomes (Fig. 1).

### 2.2. Interleukin family-mediated regulation of neuroinflammation

Interleukins (ILs) are key immunomodulatory cytokines, and an imbalance between their pro- and anti-inflammatory actions critically influences perioperative—particularly postoperative—neuroinflammation. Among them, IL-6 is a pleiotropic cytokine that signals through multiple distinct pathways (Murakami et al., 2019). Clinical studies have shown that peripheral IL-6 levels rise rapidly in the early postoperative phase and serve as a key predictor of early postoperative neurocognitive impairment (Saxena et al., 2022). Moreover, plasma IL-6 levels are strongly associated with both cognitive recovery and long-term impairment one year after major surgery in elderly patients (Taylor et al., 2023).

Elevated IL-6 and IL-1β levels have been detected in the blood, spleen, and hippocampus of mice subjected to left carotid artery exposure under anesthesia, accompanied by impaired learning and memory performance (Lai et al., 2021a). Anesthesia/surgery-induced blood–brain barrier disruption and cognitive deficits are strongly age-dependent, and systemic IL-6 knockout significantly mitigates these postoperative impairments (Yang et al., 2017; Yuan et al., 2023). In a tibial fracture model under isoflurane anesthesia, hippocampal IL-6 levels were markedly elevated. Preoperative blockade of the IL-6 receptor (IL-6R) effectively attenuated postoperative hippocampal inflammation, BBB disruption, and cognitive deficits (Hu et al., 2018). This suggests that IL-6 is necessary and sufficient for PNDs. Importantly, surgery triggered substantial infiltration of bone marrow–derived monocytes (BMMs) into the hippocampus. BMMs depletion reduced hippocampal IL-6 elevation and alleviated cognitive deficits. Notably, BMMs recruitment to the CNS appears especially pronounced following orthopedic procedures (Hu et al., 2018). Further studies revealed that IL-6–mediated cognitive dysfunction following orthopedic surgery is largely driven by non-classical trans-signaling via the co-receptor GP130, rather than classical IL-6R pathways (Hu et al., 2022), as specific deletion of IL-6Rα in hippocampal neurons or microglia failed to prevent memory deficits or STAT3 hyperphosphorylation.

IL-33, a member of the IL-1 cytokine family, is primarily secreted by astrocytes, while its receptors ST2L and IL-1RAcP are predominantly expressed on microglia and play a critical role in modulating microglial activity and broader immune responses (Liew et al., 2016; Marx et al., 2023). Under physiological conditions, astrocyte-derived IL-33 signals microglia to mediate synaptic phagocytosis, thereby maintaining synaptic homeostasis, regulating synapse number in the spinal cord and thalamus, and supporting neural circuit maturation (Vainchtein et al., 2018).

In a PND mouse model of distal tibial fracture under anesthesia, hippocampal IL-33 and ST2L expression significantly declined post-surgery, resulting in extracellular matrix accumulation and proinflammatory microglial polarization. Administration of recombinant IL-33 reversed these effects, restoring long-term potentiation and improving cognitive function (Li et al., 2023a). In contrast, in a PND mouse model induced by exploratory laparotomy, hippocampal IL-33 and ST2L expression increased three days post-surgery, potentially reflecting differences in surgical type or timing. Postoperative IL-33 administration similarly enhanced dendritic spine density and synaptic plasticity in CA1 neurons, ameliorating cognitive deficits. However, despite increasing microglial number and phagocytic activity, IL-33 treatment also altered microglial morphology and accelerated microglial dystrophy (Yang et al., 2024a). Therefore, the precise role of IL-33 in preserving synaptic homeostasis after surgery remains to be fully elucidated.

**Fig. 2.**
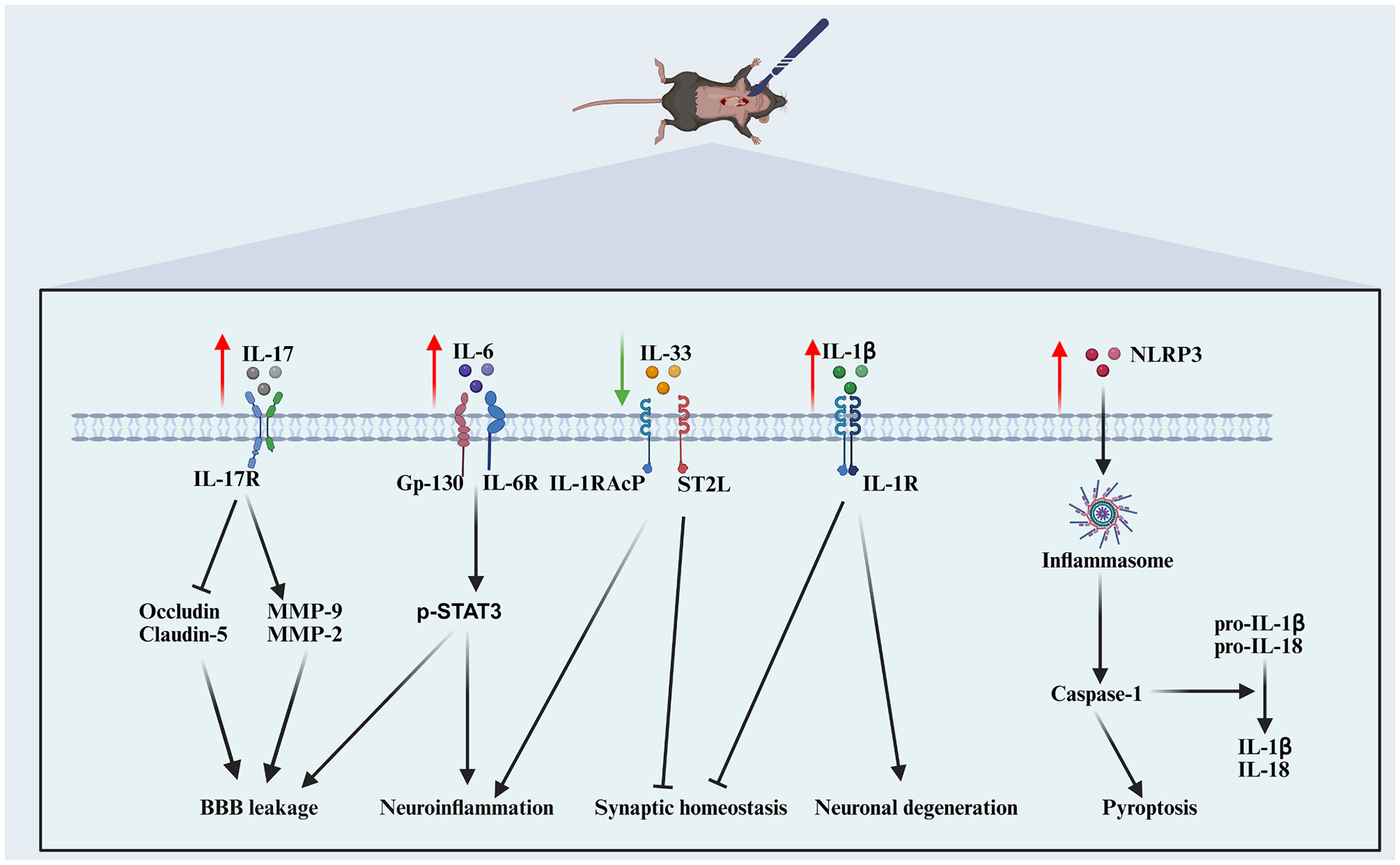
Interleukin- and NLRP3-mediated mechanisms in postoperative neuroinflammation and cognitive impairment. Anesthesia and surgery upregulate IL-6, IL-1β, IL-17A, and NLRP3 while downregulating IL-33, engaging distinct receptor pathways to disrupt BBB integrity, activate p-STAT3 signaling, impair synaptic homeostasis, and induce neuronal degeneration. BMMs infiltration into the hippocampus promotes IL-6 elevation, sustaining neuroinflammation. IL-17A suppresses tight junction proteins and elevates MMP-2/9, leading to BBB leakage. IL-33 regulates synaptic plasticity via microglial modulation, with bidirectional effects depending on surgical context. IL-1β from microglia-derived exosomes directly drives neuronal and synaptic loss. NLRP3 inflammasome activation triggers caspase-1–dependent maturation of IL-1β/IL-18 and pyroptosis, amplified by peripheral immune cell infiltration, contributing to postoperative cognitive deficits.

In addition, elevated expression of the proinflammatory cytokines IL-1β and IL-17A was detected in fracture and exploratory laparotomy models, respectively. Notably, IL-1β overexpression in M1 microglia–derived exosomes directly induced hippocampal neuronal degeneration and synaptic loss after surgery (Qi et al., 2023). Following fracture surgery, IL-17A and its receptor are upregulated in both serum and hippocampus, suppressing tight junction proteins occludin and claudin-5 while enhancing matrix metalloproteinase (MMP)-2 and MMP-9 expression. This disrupts BBB integrity and contributes to post-operative memory impairment (Ni et al., 2018). Selective inhibition of MMP-2 and MMP-9 by SB-3CT can rescue the BBB damage and neuroinflammation caused by surgery and improved postoperative cognition (Ji et al., 2023) (Fig. 2).

In summary, the interleukin family exerts diverse effects on post-operative neuroinflammation–related cognitive impairment and represents a promising target for therapeutic intervention.

### 2.3. NLRP3-mediated inflammasome activation and pyroptosis

NOD-like receptor protein 3 (NLRP3) is a key innate immune sensor that detects both exogenous pathogens and endogenous cellular damage (Mangan et al., 2018). Upon sensing danger signals, NLRP3 forms inflammasomes that activate caspase-1, driving the maturation and release of IL-1β and IL-18 and triggering pyroptotic cell death (Fu and Wu, 2023). In an isoflurane-induced PND mouse model, aged mice exhibited increased hippocampal expression of NLRP3 and caspase-1 along with cognitive deficits, whereas young mice showed no such changes (Wang et al., 2018a). Similarly, in a sevoflurane-induced cognitive impairment model in aged mice, reactive oxygen species (ROS) triggered NLRP3 activation, leading to pyroptosis of hippocampal microglia (Zhou et al., 2023a). Another study reported that in a fracture-induced PND mouse model, astrocytes in the hippocampal CA1 region showed significant upregulation of NLRP3, IL-18, IL-1β, and caspase-1. Astrocyte-specific NLRP3 knockout reversed surgery-induced impulsivity and cognitive deficits in aged mice (Wang et al., 2023a).

As noted above, fracture-induced PND models exhibit substantial infiltration of BMMs into the CNS, promoting neuroinflammation. Similarly, in a thoracic surgery mouse model, elevated peripheral Ly6C^hi^ monocytes triggered learning deficits and cortical synaptic dysfunction via NLRP3/IL-1β pathway activation (Chen et al., 2022a). Surgery did not directly activate cortical inflammasomes or induce caspase-1–mediated apoptosis or pyroptosis, whereas depletion of peripheral monocytes or treatment with the NLRP3 inhibitor MCC950 effectively rescued cortical neuronal damage and synaptic deficits (Chen et al., 2022a). Stroke mice constructed during the perioperative period of intestinal resection showed more severe cognitive deficits than stroke mice that did not undergo surgery (Zhang et al., 2022a). CD8^+^ T lymphocyte invasion into the brain parenchyma and neuroinflammation aggravated cerebral ischemic injury. The immunometabolite S-2-hydroxyglutarate promoted CD8^+^ T lymphocyte proliferation and differentiation and exacerbated CD8^+^ T lymphocyte-mediated neurotoxicity (Zhang et al., 2022a). Therefore, the activation, migration, infiltration and metabolism of major immune cells in the surgical site may be an important mechanism of CNS inflammation (Fig. 2).

### 2.4. Other cytokine-mediated neuroinflammation in PNDs

PNDs are increasingly recognized to involve multifactorial neuroinflammatory cascades beyond the interleukin family. Tumor necrosis factor-alpha (TNF-α) is one of the most consistently elevated proinflammatory cytokines observed in PNDs, both in the peripheral circulation and the central nervous system (Hirsch et al., 2016; Fertleman et al., 2022). In elderly patients undergoing hip replacement, cerebrospinal fluid levels of TNF-α are significantly higher in those who develop PNDs compared to non-PNDs counterparts (Guo et al., 2025). TNF-α was found as an upstream regulator of IL-1, and its blockade effectively limits IL-1β release, thereby preventing neuroinflammation and PNDs (Terrando et al., 2010). Recent studies have shown that surgery-induced elevation of TNF-α is associated with dysfunctional regulatory T cells (Tregs) in aged mice. CD25 blockade reduces IL-2 signaling in aged Tregs, thereby lowering hippocampal TNF-α levels and restoring blood–brain barrier integrity (Zhou et al., 2023b). However, the intrinsic mechanisms by which dysfunctional Tregs modulate microglial TNF-α production remain to be elucidated.

High-mobility group box 1 (HMGB1) is not a classical cytokine but a key damage-associated molecular pattern. Sterile surgical trauma triggers HMGB1 release, which binds to pattern recognition receptors on bone marrow-derived macrophages (BM-DM), initiating innate immune responses that contribute to postoperative cognitive decline (Vacas et al., 2014). Preoperative blockade of HMGB1 or depletion of BM-DM effectively prevents postoperative memory impairment. In addition, HMGB1 is a key activator of the Toll-like receptor 4 (TLR4)/nuclear factor κB (NF-κB) signaling pathway (Muscat et al., 2023). TLR4 signaling exacerbates MMP/tissue inhibitor of metalloproteinase (TIMP) imbalance, disrupts the blood–brain barrier, and promotes sustained inflammatory cytokine release, thereby aggravating PNDs (Zhang et al., 2020a). TLR2 is also upregulated in the hippocampus following carotid artery exposure in mice, concomitant with HMGB1 activation, leading to neuroinflammation and cognitive impairment (Lin et al., 2021a). Inhibiting HMGB1 or reducing surgery-induced infiltration of BM-DM represents a promising strategy for preventing PNDs.

Chemokines are critical mediators of neuroinflammation in PNDs, orchestrating immune cell recruitment and amplifying inflammatory signaling within the central nervous system. Following exploratory laparotomy in mice, hippocampal expression of C-X-C motif chemokine (CXCL) 13 and its receptor CXCR5 is upregulated, promoting ERK phosphorylation and the production of IL-1β and TNF-α (Shen et al., 2020). Inhibition of either CXCL13 or CXCR5 effectively rescues post-operative cognitive deficits. Elevated serum levels of CCL2 have also been observed in patients undergoing carotid endarterectomy and are associated with acute postoperative neurocognitive dysfunction (Mack et al., 2008).

Recent studies have revealed that surgery-induced neuroprotective factors also play a direct role in modulating neuroinflammation. Propofol exposure reduces hippocampal expression of nerve growth factor (NGF) and cAMP response element-binding protein (CREB) in mice, suppresses CREB phosphorylation, and induces M1 polarization of microglia, thereby promoting the progression of neuroinflammation (Ye et al., 2025). Nuclear factor erythroid 2–related factor 2 (Nrf2), a key neuroprotective factor, is also significantly downregulated in the hippocampus of mice with sevoflurane-induced cognitive impairment. Pre-injection of Nrf2-expressing adeno-associated virus into the hippocampus suppresses subsequent sevoflurane-induced microglial activation and proinflammatory cytokine release in a dose-dependent manner (Li et al., 2025). Similarly, mesencephalic astrocyte-derived neurotrophic factor (MANF) has been shown to attenuate sevoflurane-induced cognitive impairment. Unlike Nrf2, sevoflurane exposure increases endogenous MANF expression in hippocampal microglia, which suppresses TNF-α production, neuronal apoptosis, and synaptic damage, serving as an intrinsic neuroprotective mechanism (Gao et al., 2024). These findings highlight the complex roles of neuroprotective factors in PNDs, involving both suppression and compensatory activation. Whether boosting intrinsic pathways like MANF or externally regulating factors such as Nrf2 offers more effective therapeutic potential remains an open question for future investigation.

## 3. Neurotransmitter and receptor dysfunction

Neurotransmitter and receptor dysfunction is recognized as a key pathological mechanism underlying cognitive deficits, neuropsychiatric disorders, and neurodegenerative diseases (Sarter et al., 2007). The neuron–astrocyte–mediated glutamate/GABA–glutamine cycle is essential for sustaining neurotransmission and cellular energy metabolism and plays a critical role in the pathogenesis of neurodegenerative diseases (Andersen et al., 2022). Dysfunction of dopaminergic and cholinergic systems further contributes to neurodegenerative disease progression and symptom severity (Davis et al., 2023). Personalized brain modeling and neuroimaging studies have revealed that GABAergic signaling significantly influences cognitive executive function in Alzheimer’s disease (AD), while glutamatergic and cholinergic systems modulate the distribution of amyloid-β and tau pathology (Khan et al., 2022). *In vivo* brain imaging in mice demonstrated high synchronization of neurotransmitter networks during propofol and sevoflurane anesthesia. Notably, surgical-dose propofol–induced unconsciousness was associated with significant increases in cortical GABA, glutamate, norepinephrine, acetylcholine, and dopamine levels, whereas sevoflurane anesthesia led to reductions in all five neurotransmitters (Qiu et al., 2024). Thus, anesthesia- and surgery-induced alterations in neurotransmitter systems profoundly influence the development of PNDs.

Previous studies have shown that cognitive dysfunction under general anesthesia is driven by enhanced activity of GABA type A receptors (GABA_A_Rs). Notably, α5GABA_A_Rs activity remains elevated even after anesthetic clearance, leading to impaired hippocampal memory performance and synaptic plasticity (Zurek et al., 2014). In a mouse laparotomy model following isoflurane anesthesia, surgery induced hippocampal-dependent memory deficits and impaired LTP. While anesthesia alone had no effect, the combination of anesthesia and laparotomy significantly reduced hippocampal GABA levels. Moreover, surgery upregulated α5GABA_A_Rs expression in hippocampal pyramidal neurons via the p38/MAPK pathway, enhancing GABA binding and contributing to cognitive impairment (Zhang et al., 2020b). Following surgery, hippocampal GABAergic tone declines, yet α5GABA_A_Rs expression paradoxically increases, exacerbating tonic inhibition and contributing to cognitive deficits. Within 24 h after sevoflurane anesthesia, the expression and synaptic distribution of α5GABA_A_Rs in hippocampal neurons remained dysregulated, contributing to sustained postoperative cognitive impairment (Wang et al., 2024a). Recent studies have identified myeloid differentiation factor 2 (MD2), a secreted glycoprotein involved in TLR4-mediated neuroinflammation, as a key regulator of anesthesia/surgery-induced α5GABA_A_Rs upregulation. Targeted inhibition of MD2 via viral vectors or degradation peptides reduced α5GABA_A_Rs membrane expression in hippocampal CA1 pyramidal neurons, thereby reversing surgery-induced cognitive impairment and delayed recovery (Zuo et al., 2021).

Glutamatergic neurons release the excitatory neurotransmitter glutamate into the synaptic cleft, generating excitatory postsynaptic currents. Anesthesia- and/or surgery-induced neurocognitive disorders have been linked to reduced excitability of hippocampal glutamatergic neurons and impaired synaptic plasticity (Wu et al., 2024; Wan et al., 2024; Ma et al., 2022). Activation of hippocampal glutamatergic neurons is an effective strategy for preventing PNDs. N-methyl-D-aspartate receptors (NMDARs), a subtype of ionotropic glutamate receptors, consist of the core NR1 subunit and modulatory NR2 subunits. Variations in NR2 composition confer distinct regional distribution and physiological properties, playing essential roles in neural development, learning, and memory (Liu et al., 2023a; Battaglia et al., 2023; Chen et al., 2023b). However, excessive activation of neural excitability mediated by NMDAR can also lead to neural dysfunction (Xhima et al., 2016; Kim et al., 2022). Studies have shown that neuroinflammation in PNDs model mice causes sustained downregulation of NR2A and NR2B subunits at dorsal hippocampal synapses (Chen et al., 2022b). Dendritic spine plasticity was markedly impaired in operated mice, resulting in long-term memory deficits. Transient postoperative inflammation may extend the duration of cognitive impairment by sustaining NMDAR dysfunction.

In a carotid artery exposure mouse model, surgery activated the lateral habenula (LHB)–ventral tegmental area (VTA) pathway, which projected to the hippocampus and prefrontal cortex (Xin et al., 2022). LHB-driven activation of VTA neurons led to sustained NMDAR activation, inducing endoplasmic reticulum stress and neurotoxicity. Inhibition of NMDARs in the VTA or suppression of LHB signaling mitigated ER stress and neurocognitive impairment. Additionally, blocking VTA dopaminergic projections to the hippocampus and prefrontal cortex partially rescued postoperative cognitive deficits (Xin et al., 2022). The locus coeruleus (LC)–dorsal hippocampal CA1 dopaminergic circuit plays a key role in regulating memory consolidation and cognitive function (Yamasaki and Takeuchi, 2017; Chowdhury et al., 2022). In contrast to surgery-induced VTA projections that exacerbate neurotoxicity, activation of locus coeruleus–CA1 dopaminergic pathways alleviates postoperative synaptic damage and cognitive impairment (Zhang et al., 2024a). Despite both projecting to the hippocampus and prefrontal cortex, the LHB–VTA pathway promotes postoperative neurotoxicity through sustained NMDAR activation and ER stress, whereas activation of the LC–CA1 dopaminergic circuit enhances synaptic plasticity and confers cognitive resilience.

Studies have shown that anesthesia/surgery-induced NMDAR activation not only enhances glutamatergic signaling but also facilitates Ca^2+^ influx through its ion channel function (Qiu et al., 2020). Ca^2+^ influx activates calpain, which cleaves TrkB-FL and disrupts BDNF/TrkB signaling, thereby exacerbating neurocognitive dysfunction. These findings highlight the anesthesia/surgery-induced imbalance between glutamatergic neurons and NMDAR signaling, underscoring the need for integrated strategies in future research.

Studies have shown that purinergic ionotropic receptors (P2X), which respond to extracellular ATP, aggravate neurocognitive disorders during surgery by promoting neuroinflammation and pyroptotic cell death (Yuan et al., 2022). P2X upregulation–induced microglial activation is a key pathogenic mechanism in conditions such as surgical trauma, hypoxia, amyotrophic lateral sclerosis (ALS), and Huntington’s disease (HD) (Martire et al., 2020; Chisari et al., 2023; Apolloni et al., 2021; Yang et al., 2024a). Decreased levels of α7 nicotinic acetylcholine receptor (α7nAChR) in the hippocampus after surgery exacerbate neuroinflammation and block the neuroprotective effects of the BDNF/TrkB signaling pathway (Terrando et al., 2015; Wei et al., 2022). As α7nAChR is permeable to Ca^2+^, impaired Ca^2+^ influx through this receptor may disrupt intracellular signaling and neurotransmitter release, thereby contributing to the development of PNDs (Noviello et al., 2021).

In summary, anesthesia and surgery induce substantial disruptions in neurotransmitter and receptor systems. Further studies are needed to clarify the specific roles of neurotransmitter receptor dysfunction in the pathogenesis of PNDs (Fig. 2).

## 4. Epigenetic modifications

### 4.1. DNA and histone methylation

Epigenetic modification is a key mechanism for investigating gene function changes under external stimuli, with DNA methylation being one of the most prevalent forms. By restricting chromatin accessibility to cis-regulatory elements, DNA methylation induces gene silencing (Pacis et al., 2019). Hypermethylation of CpG sites in multiple synaptic genes has been observed in the prefrontal cortex of aged mice, closely linked to aging and age-related cognitive decline (Ianov et al., 2017).

Following anesthesia exposure, aged mice exhibited reduced hippocampal mRNA and protein levels of Arc, BDNF, and RELN, accompanied by increased expression of DNA methyltransferases (DNMTs) and methyl-CpG-binding protein 2 (MECP2) (Ni et al., 2020; Ju et al., 2016; Wu et al., 2016). Sevoflurane increased promoter methylation of Arc, BDNF, and RELN, suppressing their transcription. Treatment with DNA methyltransferase inhibitors or noninvasive environmental enrichment reversed the anesthesia-induced downregulation of memory-related genes and improved postoperative cognitive outcomes. Additionally, abdominal exploratory surgery in anesthetized mice induced H3K9 trimethylation, which enhanced binding to the BDNF exon IV promoter, leading to reduced BDNF expression and memory impairment (Wu et al., 2019). Moreover, in a neonatal mouse model of sevoflurane exposure, hypermethylation of multiple synaptic genes, including Shank2, Psd95, Syn1, and Syp, was observed, resulting in reduced hippocampal synaptic protein levels and decreased synaptic density (Fan et al., 2021a; Shi et al., 2023). Pretreatment with methyltransferase inhibitors attenuated synaptic gene hypermethylation and restored learning and spatial memory in anesthesia-exposed mice.

As mentioned above, anesthesia and surgery led to decreased expression of the NMDAR regulatory subunit NR2 (Chen et al., 2022b). Targeted bisulfite sequencing revealed hypermethylation at multiple sites surrounding the NR2 promoter, suggesting epigenetic suppression of NMDAR subunit expression (Xu et al., 2023). Furthermore, sevoflurane exposure in adult mice increased methylation of the glucocorticoid receptors (GRs) exon 17 promoter in the hippocampus, indicating stress-related epigenetic alterations (Zhu et al., 2017). Reduced GRs gene expression exacerbates anesthesia-induced neuroinflammation and cognitive impairment.

**Fig. 3.**
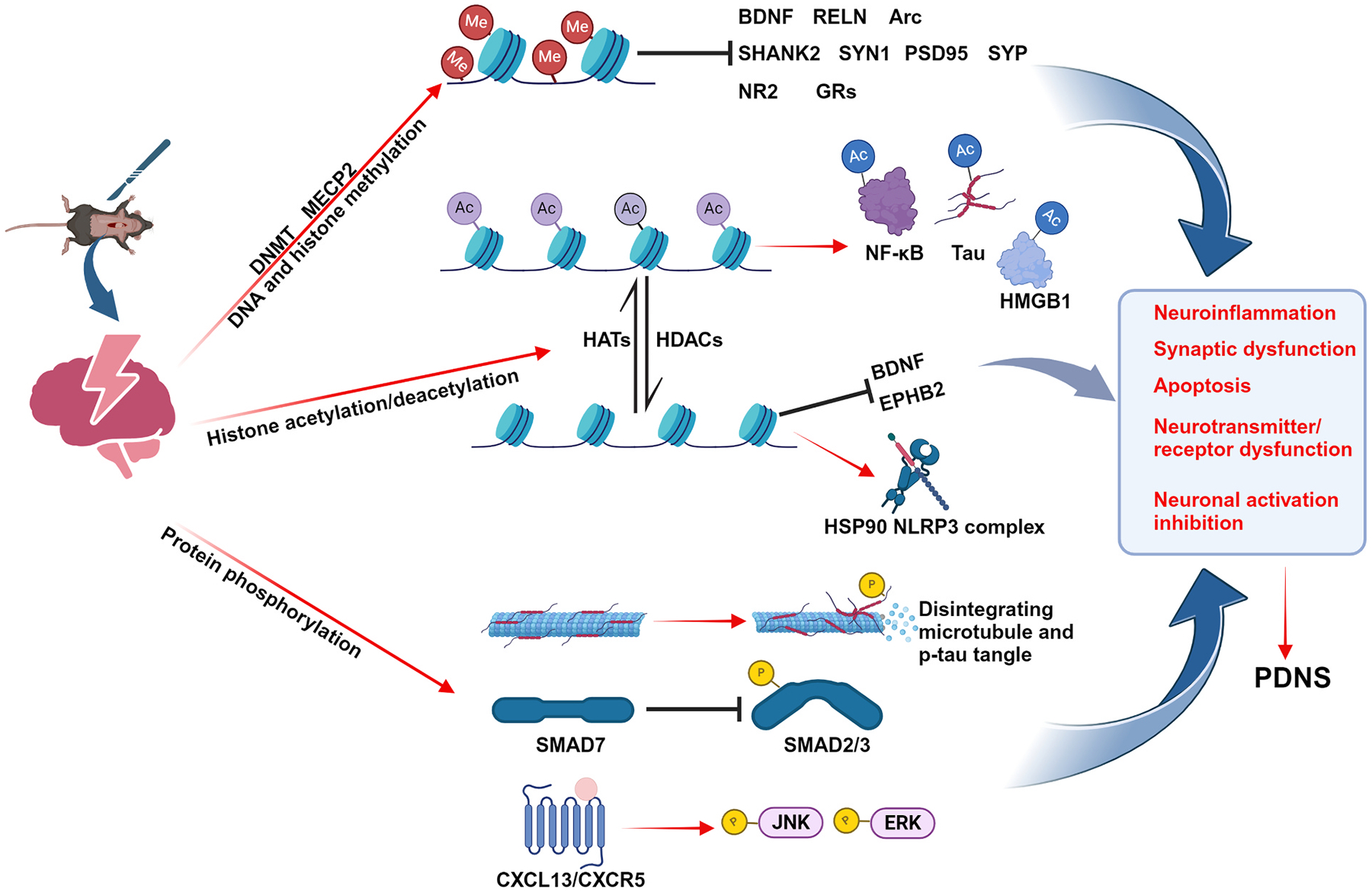
Epigenetic modifications participate in the regulation of PNDs. Anesthesia/surgery promotes the recruitment of DNMT and MECP2, promoting DNA methylation and inhibiting the expression of neurodevelopmental factors, synaptic proteins, and neurotransmitter receptors. HATs mediate acetylation of NF-κB, Tau, and HMGB1, exacerbating neural damage, and HDACs mediate deacetylation of BDNF and EPHB2, promoting neurocognitive disorders. HSP90 deacetylation promotes binding to NLRP3 and enhances NLRP3 protein stability and resistance to proteolysis. Surgery induces microtubule disintegration and tau phosphorylation, and the ectopic distribution of free p-tau exacerbates neuronal damage. Overexpression of smad7 inhibits smad2/3 phosphorylation and hinders the anti-inflammatory effect of the downstream TGF-β/TGF-βR signaling pathway. CXCL13/CXCR5 signaling activation promotes ERK/JNK phosphorylation, exacerbating neuroinflammation and cell apoptosis.

Interestingly, while many studies have demonstrated that anesthesia and surgery promote cognitive impairment through increased DNA methylation and suppression of memory-associated genes, recent evidence suggests a more complex, age-dependent epigenetic landscape. A study using a laparotomy-induced PNDs model in aged mice revealed a significant reduction of DNMT3a in hippocampal neurons, leading to global hypomethylation. This loss of DNMT3a function resulted in the derepression of leucine-rich alpha-2-glycoprotein 1 (LRG1), which activated TGF-β signaling and contributed to synaptic dysfunction and cognitive deficits. Notably, restoring DNMT3a expression or silencing LRG1 reversed these pathological changes (Cong et al., 2025). Although both studies employed aged mouse models, the opposing roles of DNMT3a suggest that factors beyond age, such as surgical type, anesthesia method, cell specificity, and timing of epigenetic changes, may critically influence the direction of DNA methylation dynamics. These findings underscore the complexity of epigenetic regulation in PNDs and the importance of context-specific investigation (Fig. 3).

### 4.2. Histone acetylation

Histone acetylation is a well-characterized post-translational modification, in which histone acetyltransferases (HATs) transfer acetyl groups from acetyl-CoA to lysine residues on histone tails, altering chromatin structure and gene expression (Tanner et al., 2000; Gao et al., 2021a; White et al., 2024). Histone acetylation is a reversible process, with histone deacetylases (HDACs) removing acetyl groups to counter-balance HAT activity. Unlike DNA methylation–mediated gene silencing, histone acetylation promotes transcription by facilitating the recruitment of transcription factors and RNA polymerase II (Maity et al., 2021). Both histone acetylation and deacetylation play critical roles in brain function. During neurodevelopment, histone acetyltransferases enhance the transcriptional responsiveness of neuronal gene networks, such as immediate early genes, thereby facilitating memory consolidation in mice (Mews et al., 2017; Li et al., 2021b). This epigenetic regulation is particularly relevant in the context of PNDs, where disruptions in histone acetylation balance may underlie postoperative cognitive decline.

As noted above, anesthesia and surgery upregulate MeCP2 expression in the mouse hippocampus, enhancing its interaction with DNMT1 and promoting BDNF promoter methylation, thereby reducing BDNF expression (Wu et al., 2016). In addition, the experiment also observed an increase in HDAC2 expression in the CA1 region of the hippocampus. The enhanced interaction between HDAC2 and MeCP2 reduced the occupancy of acetylated H3 histones in the BDNF promoter region, thereby leading to reduced BDNF expression (Wu et al., 2016). Therefore, multiple epigenetic modifications induced by anesthesia and surgery have remarkable spatiotemporal consistency and influence each other.

EphrinB2 receptor (EPHB2R) is a key regulator of NMDAR trafficking and synaptic localization. EPHB2 deficiency results in depression-like behavior and cognitive impairment in mice, whereas EPHB2 overexpression restores synaptic NMDAR targeting and rescues memory deficits in AD models (Mikasova et al., 2012; Hu et al., 2017; Zhen et al., 2018). In an isoflurane-exposed mouse model, reduced EPHB2 expression was accompanied by impaired phosphorylation and trafficking of GluN2B-containing NMDARs. Surgery markedly increased HDAC2 enrichment at the EPHB2 promoter, suppressing its transcription. HDAC2 knockdown restored EPHB2-dependent NMDAR trafficking by enhancing histone acetylation (Hao et al., 2023).

Tau acetylation is markedly increased in the cortex and hippocampus following traumatic brain injury in mice, contributing to axonal degeneration and aberrant tau localization. Inhibition of tau acetylation alleviates neurodegenerative pathology and cognitive deficits (Shin et al., 2021; Parra Bravo et al., 2024). In a laparotomy mouse model under anesthesia, neuroinflammation downregulated the expression of the deacetylase Sirtuin 1 (Sirt1), resulting in increased acetylation of NF-κB p65 (Jiang et al., 2023). In a splenectomy-induced PND model, hippocampal neuronal expression of HDAC4/5 was significantly reduced, leading to increased acetylation of high mobility group box 1 (HMGB1) (Huang et al., 2021). HMGB1 is a structural protein of chromatin that is involved in neuroinflammation in neurodegenerative diseases and PNDs through autoimmunity and phagocytosis (Terrando et al., 2016; Tang et al., 2023b; Zhang et al., 2020c). However, the splenectomy model showed elevated HDAC6 expression in the hippocampus, resulting in increased deacetylation of heat shock protein 90 (HSP90) (Lin et al., 2024). HSP90 deacetylation strengthens its binding to NLRP3, impedes NLRP3 degradation, and exacerbates surgery-induced neuroinflammation and pyroptosis. While acetylation of pathological proteins induced by brain trauma and surgery contributes to neural injury, the differential expression of HDACs in surgery-related cognitive impairment points to distinct and context-specific regulatory mechanisms (Fig. 3).

### 4.3. Protein phosphorylation

Protein phosphorylation, catalyzed by protein kinases, is one of the most prevalent and functionally significant post-translational modifications. Numerous studies have shown that anesthesia and surgery induce tau hyperphosphorylation in the hippocampus of young mice, contributing to heightened neuroinflammation and cognitive deficits (Tao et al., 2014; Liang et al., 2023). In the healthy brain, tau stabilizes microtubules and supports axonal transport. In contrast, in AD, hyperphosphorylated tau accumulates and dissociates from microtubules, disrupting neuronal function (Drummond et al., 2020). Sevoflurane induces sustained tau phosphorylation at multiple hippocampal sites, promoting tau dissociation from microtubules and elevating free tau levels. Recent studies further reveal that anesthesia-induced tau hyperphosphorylation enhances excitatory input from the hippocampal CA1 region to anterior cingulate cortex (ACC) pyramidal neurons, while inhibition of tau phosphorylation attenuates ACC hyperactivity and mitigates anesthesia-related cognitive deficits (Chen et al., 2024a). Tau acetylation and phosphorylation play critical roles in neurocognitive dysfunction and represent promising therapeutic targets for preventing PNDs.

Protein phosphorylation also initiates various anesthesia/surgery-induced signaling pathways, including neuroinflammation, autophagy, and pyroptosis. In a unilateral nephrectomy mouse model under anesthesia, Smad7 expression was markedly elevated in the hippocampal CA1 region, suppressing Smad2/3 phosphorylation (Liu et al., 2023a). The phosphorylated Smad2/3–Smad4 heterocomplex is essential for mediating the anti-inflammatory signaling cascade of the TGF-β/TGF-βR pathway (Di Fusco et al., 2017). Smad7 blockade or knockout restored Smad2/3 phosphorylation, thereby attenuating surgery-induced neuroinflammation and cognitive impairment.

c-Jun N-terminal kinase (JNK), a central component of the mitogen-activated protein kinase (MAPK) cascade, plays a pivotal role in mediating perioperative neuroinflammation and pyroptotic cell death (Zhang et al., 2019). In the DNR mouse model, microglial JNK phosphorylation was markedly increased, activating NLRP3 inflammasome signaling and inducing pyroptotic cell death (He et al., 2021). LPS-induced JNK phosphorylation was not attenuated by NLRP3 inhibition, suggesting that JNK may function upstream of NLRP3 activation. Similarly, extracellular signal–regulated kinase (ERK), another key MAPK pathway component, is implicated in both brain development and neuropathological processes (Iroegbu et al., 2021; Zhou et al., 2019). Studies have shown that ERK phosphorylation is regulated by chemokine signals (Guo et al., 2023; Wu et al., 2022). In the exploratory laparotomy PNDs model, hippocampal expression of CXCL13 and its receptor CXCR5 was significantly upregulated. This CXCL13/CXCR5 signaling enhanced ERK phosphorylation, promoting the release of IL-1β and TNF-α and exacerbating neuroinflammation (Shen et al., 2020). Knockout of CXCL13 or CXCR5 suppresses ERK phosphorylation and mitigates surgery-induced cognitive impairment.

Studies have shown that anesthesia-induced upregulation and mislocalization of α5GABA_A_Rs in the hippocampus of aged mice are linked to activation of the phosphorylated p38/MAPK signaling pathway (Zhang et al., 2020b; Wang et al., 2024a). The synaptic distribution of α5GABA_A_Rs is controlled by radixin phosphorylation, which is regulated by the RhoA/ROCK signaling pathway (Hausrat et al., 2015; Loebrich et al., 2006). Sevoflurane exposure significantly activated RhoA/ROCK signaling in the hippocampus, resulting in increased expression of α5GABA_A_Rs and phosphorylated radixin (Loebrich et al., 2006). Phosphorylated radixin-mediated excessive extrasynaptic anchoring of α5GABA_A_Rs contributes to cognitive impairment in mice. These findings further underscore the critical role of protein post-translational modifications in regulating neurotransmitter receptor function (Fig. 3).

### 4.4. Functional noncoding RNA regulation

**Fig. 4.**
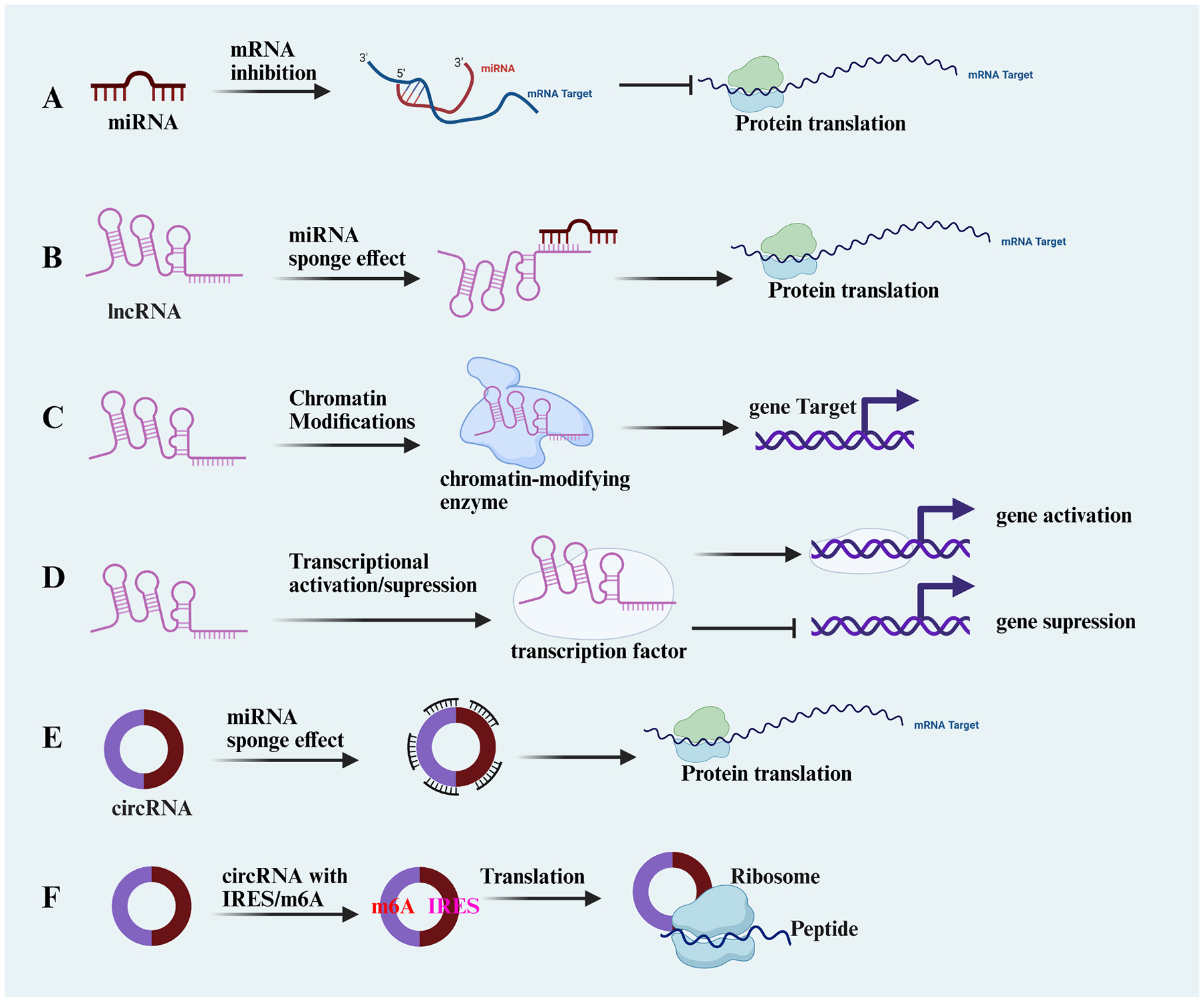
Regulatory mechanisms of miRNAs, lncRNAs, and circRNAs. (A) miRNAs bind to target mRNAs to inhibit translation. (B–D) lncRNAs regulate gene expression via miRNA sponging, chromatin modification, or transcription factor modulation, leading to gene activation or suppression. (E–F) circRNAs act as miRNA sponges or, when containing IRES/m6A elements, directly undergo translation into peptides.

Non-coding RNA (ncRNA) refers to functional RNA molecules that cannot be translated into proteins and are part of epigenetic modifications. Common ncRNAs with regulatory functions include small interfering RNA (siRNA), microRNA (miRNA), PIWI-interacting RNA (piRNA), circular RNA (circRNA) and long non-coding RNA (lncRNA). NcRNAs can directly regulate chromatin structure and can also interact with other mechanisms to co-regulate epigenetic marks (Wang et al., 2022a; Wu et al., 2023). Accumulating evidence indicated that ncRNA-mediated epigenetic modifications are involved in the progression of PNDs (Fig. 4).

Microarray analysis of hippocampal tissue from a tibial fracture–induced PND model revealed that miR-181b-5p was the most significantly downregulated miRNA (Lu et al., 2019). Both *in vivo* and *in vitro* studies demonstrate that miR-181b-5p suppresses surgery-induced neuroinflammation and LPS-triggered microglial activation. Dual-luciferase assays confirmed that miR-181b-5p directly targets the 3′-UTR of TNF-α, inhibiting its transcription. Stereotaxic hippocampal injection of a miR-181b-5p agonist in PNDs mice reduced neuroinflammation and improved cognitive function (Lu et al., 2019). Similarly, reduced hippocampal expression of miR-124–3p in PNDs mice led to upregulation of its target gene LPIN1, a regulator of immune responses and inflammation, thereby exacerbating surgery-induced neuroinflammation and neuronal apoptosis (Han et al., 2021). In addition, miR-214–3p levels increased in the peripheral circulation but decreased in the hippocampus of rats following surgery (Wang et al., 2022b). Both preoperative and postoperative administration of miR-214–3p suppressed its target gene prostaglandin-endoperoxide synthase 2 (PTGS2) and attenuated postoperative cognitive impairment. MiRNA-let-7b is a highly conserved and brain-enriched miRNA that activates the TLR7 signaling pathway in macrophages and microglia, contributing to neurodegeneration (Lehmann et al., 2012). Studies have confirmed that surgery amplifies the miRNA-let-7b/TLR7 signaling axis in the mouse hippocampus, intensifying inflammatory cytokine expression and worsening memory impairment (Deng et al., 2024a).

In addition to downregulating anti-inflammatory miRNAs, surgery upregulated several miRNAs whose altered expression exerted diverse effects on the development of PNDs. In mice with tibial fracture, hippocampal miR-146a expression was upregulated, leading to suppression of its target genes, interleukin-1 receptor–associated kinase 1 (IRAK1) and TNF receptor–associated factor 6 (TRAF6) (Chen et al., 2019). MiR-146a overexpression attenuated surgery-induced hippocampal inflammation and memory deficits, whereas its knockdown exacerbated postoperative pathology. LIM kinase (LIMK), a critical regulator of actin dynamics, plays a key role in synaptic plasticity and memory formation (Ben Zablah et al., 2021). In anesthesia-induced neurocognitive impairment, increased expression of miRNA-106a and miRNA-125b-5p in the hippocampus inhibited the expression of the target gene LIMK1 and aggravated cell apoptosis and neuroinflammation (Zhang et al., 2020a; Xiong et al., 2019). Recent studies have found that astrocyte-derived exosomes carrying miRNA-26a-5p can alleviate long-term anesthesia-induced neurocognitive impairment (Li et al., 2024a). These findings collectively highlight the dual roles of miRNAs as both protective and pathogenic regulators in PNDs, raising the intriguing possibility that targeted modulation of specific miRNAs may serve as a novel therapeutic strategy.

LncRNAs primarily regulate miRNA function and expression by directly binding to miRNAs or acting as competitive endogenous RNAs (ceRNAs) (Mao et al., 2021a). In an anesthesia-induced PND mouse model, hippocampal lncRNA-Rian expression was significantly reduced. Acting as a ceRNA, this reduction led to increased miR-143–3p levels, which in turn suppressed its target gene LIMK1, aggravating anesthesia-induced neuronal damage and cognitive deficits (Yu et al., 2021). Similarly, surgery reduced hippocampal expression of lncRNA maternally expressed gene 3 (lnc-MEG3), which negatively regulated the miR-106a-5p/SIRT3 axis, contributing to postoperative neuropathology (Ye et al., 2023). In PND models, neuroinflammation or injury can also activate protective signaling pathways. In a liver ischemia–reperfusion model, hippocampal expression of lncRNA nuclear enriched transcript 1 (lnc-NET1) was upregulated, competitively binding miR-122–5p and thereby enhancing Wnt1 transcriptional activity (Dong et al., 2023). Activation of the Wnt1/β-catenin signaling pathway contributes to the attenuation of surgery-induced hippocampal injury and cognitive deficits.

Similar to lncRNAs, circRNAs function as ceRNAs, acting as molecular sponges to sequester miRNAs and relieve their repression of target gene expression (Mao et al., 2021b). Whole transcriptome sequencing of hippocampal tissue from mice undergoing tibial fracture fixation revealed a marked reduction in circRNA-AKT3 expression, which was associated with increased neuronal apoptosis and cognitive deficits (Wang et al., 2024b). Hippocampal overexpression of circRNA-AKT3 reversed neuronal apoptosis and cognitive impairment, confirming its neuroprotective role in PNDs. Unlike the classical ceRNA mechanism, circRNA-AKT3 overexpression enhanced both the binding and expression of miR-106a-5p. Surgery-induced downregulation of the circRNA-AKT3/miR-106a-5p axis increased HDAC4 expression, which strengthened its interaction with myocyte enhancer factor 2 C (MEF2C) and suppressed MEF2C activity. Reduced MEF2C transcription subsequently contributed to neuronal apoptosis and synaptic plasticity–dependent memory deficits (Zhou et al., 2023c; Udeochu et al., 2023). Currently, finding ncRNAs with high sensitivity and efficacy is an important direction for studying PNDs (Table 1).

## 5. Intestinal dysbacteriosis

**Fig. 5.**
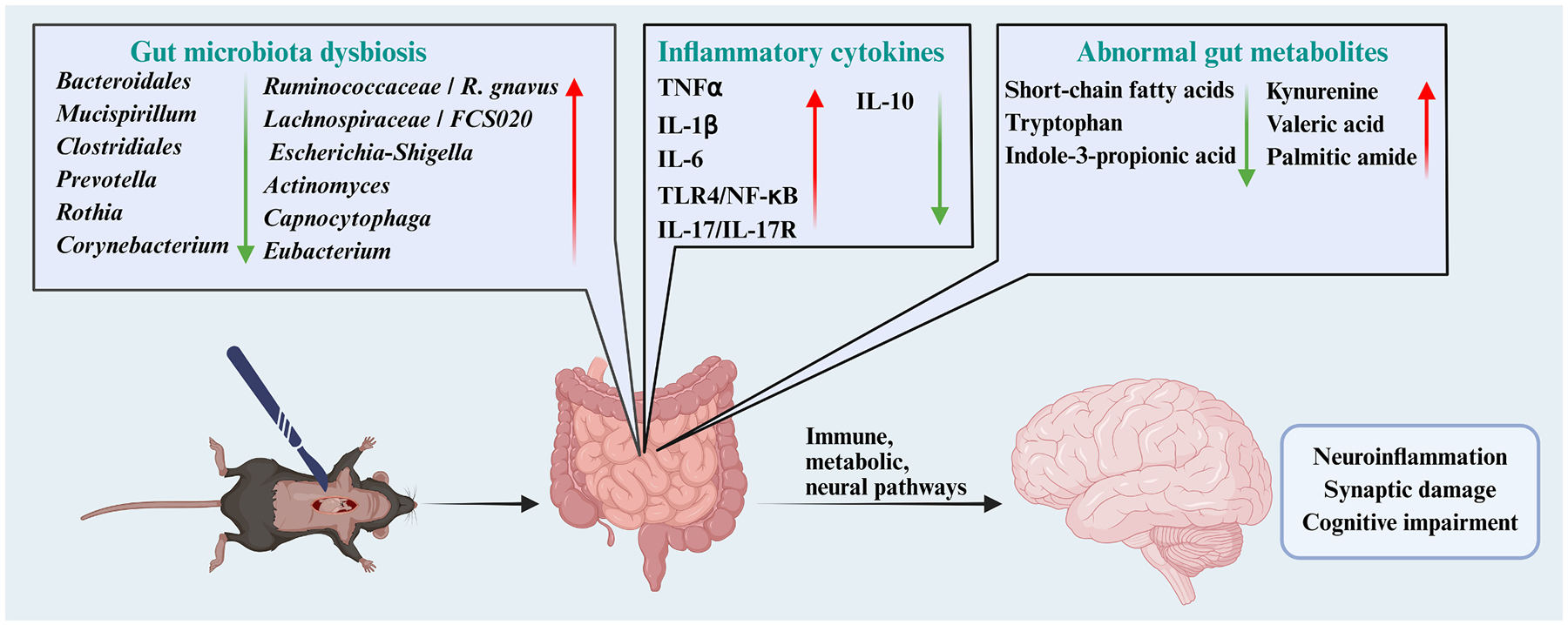
Gut microbiota dysbiosis–mediated mechanisms in PNDs. Anesthesia and surgery induce gut microbiota dysbiosis, characterized by reduced abundance of taxa such as *Bacteroidales*, *Mucispirillum*, and *Clostridiales*, and increased abundance of *Ruminococcaceae* and *Escherichia-Shigella*. These changes are accompanied by elevated proinflammatory cytokines and decreased IL-10, as well as metabolic disturbances, including reduced short-chain fatty acids, tryptophan, and indole-3-propionic acid, and increased kynurenine, valeric acid, and palmitic amide. Through immune, metabolic, and neural pathways, these alterations exacerbate neuroinflammation, synaptic damage, and cognitive impairment.

Extensive studies have confirmed a complex bidirectional regulatory network among the gut, microbiota, and brain, mediated through well-defined molecular pathways including immune, neural, and endocrine signaling (Mayer et al., 2022; Wang et al., 2023b; Agirman et al., 2021). Anesthesia/surgery and postoperative recovery are closely related to the function of intestinal microbes (Hajjar et al., 2023; Liu et al., 2023b; Minerbi and Shen, 2022). Gut microbial function has become an important mechanism that cannot be ignored for PNDs (Fig. 5). Aged mice subjected to tibial fracture or abdominal surgery under anesthesia exhibited marked disruptions in gut microbiota, with eight significantly altered bacterial taxa restored by preoperative mixed probiotic treatment (Lian et al., 2021; Jiang et al., 2019). Additionally, anesthesia disrupted the intestinal barrier and elevated serum inflammatory cytokines, thereby exacerbating neuroinflammation and cognitive impairment (Zhao et al., 2023). Preoperative dietary restriction ameliorated surgery-induced gut microbiota dysbiosis and neuroinflammation, thereby alleviating postoperative cognitive dysfunction (Ren et al., 2024). This is consistent with the clinical practice of fasting before surgery. Preoperative gut microbiota from PNDs patients induces cognitive impairment and systemic inflammation in recipient rats, both before and after surgery, with elevated TNF-α levels and microglial activation closely associated with increased *Desulfobacterota* abundance (Wei et al., 2024). It highlights the specificity of the gut microbiota as an upstream factor that increases the risk of developing PNDs.

Gut microbiota–mediated metabolic disturbances are key contributors to PNDs. In a partial hepatectomy mouse model, levels of short-chain fatty acids (SCFAs) were significantly reduced, resulting in heightened neuroinflammation and cognitive deficits (Xu et al., 2021; Dai et al., 2024). Surgery-induced dysregulation of SCFA metabolism is associated with an imbalance in histone acetylation/deacetylation (Liu et al., 2024b). Preoperative oral supplementation with SCFAs significantly mitigated surgery-induced pathological changes. Additionally, intestinal probiotics alleviated brain injury and cognitive impairment following extracorporeal circulation surgery by modulating the kynurenine metabolic pathway (Zhang et al., 2024b).

Mice undergoing left carotid artery exposure under anesthesia showed reduced gut microbiota diversity, pronounced neuroinflammation, and significant cognitive deficits (Lai et al., 2021b). Fecal transplantation from these mice into non-surgical controls induced similar learning and memory impairments. Further analysis revealed elevated circulating valeric acid levels, a gut-derived metabolite, implicating its potential role in the pathogenesis of postoperative cognitive dysfunction (Lai et al., 2021b). Preoperative plasma levels of indole-3-propionic acid (IPA), a neuroprotective gut-derived indole metabolite, were inversely correlated with the incidence of postoperative cognitive impairment in mice (Zhou et al., 2023d). IPA administration reduced the incidence of postoperative delirium. Fecal transplantation from young mice or intervention with *Lactobacillus* or IPA attenuated postoperative cognitive impairment in aged mice. Metabolomic analysis revealed that surgery elevated serum palmitic amide levels in aged mice, while fecal transplantation reduced palmitic amide concentrations in both serum and brain tissue (Pan et al., 2023). While these studies underscore the gut microbiota as a critical modulator of PNDs via metabolic pathways, current evidence remains largely correlative and model specific. The causal relationships, specific microbial taxa, and metabolite thresholds required for cognitive protection are not yet fully elucidated. Furthermore, the consistency and safety of microbiota-targeted interventions across diverse surgical contexts remain uncertain, highlighting the need for rigorous mechanistic and translational investigations.

Immune signaling is a key component of gut–brain axis communication. In an abdominal exploratory surgery model, mice exhibited gut dysbiosis accompanied by a significant increase in T helper 17 (Th17) cell levels in intestinal, circulatory, and brain tissues (Wen et al., 2022). TH17 cells activation promoted IL-17/IL-17R signaling and aggravated hippocampal inflammation. Repeated sevoflurane exposure aberrantly activates NLRP3 inflammasome signaling in both the intestine and hippocampus of mice (Han et al., 2024). Probiotic pretreatment of mice restored the abundance of *Akkermansia* and reduced hippocampal microglial inflammatory activation and synaptic damage.

Recent studies have highlighted the enteric nervous system (ENS) as a key regulator of sensory transmission, neural circuitry, and inflammation (Yan et al., 2021; Schneider et al., 2023). Whether the ENS contributes to the pathogenesis of PNDs remains an important and promising area for future investigation.

## 6. Mitochondrial homeostasis

Mitochondrial energy production is essential for sustaining CNS function. As highly dynamic organelles, mitochondria maintain homeostasis through coordinated regulation of energy metabolism, dynamics, and quality control mechanisms (Han et al., 2023; Borbolis et al., 2023). Mitochondrial dysfunction is a hallmark of PND-related neurodynamic disturbances. Mitofusin 2 (MFN2) is a key mediator of endoplasmic reticulum–mitochondria coupling, crucial for preserving mitochondrial integrity and cellular homeostasis (Naón et al., 2023). MFN2 ablation results in excessive ER–mitochondria contact, leading to mitochondrial Ca^2+^ overload and triggering apoptotic cell death (Filadi et al., 2015). Repeated sevoflurane exposure in neonatal mice significantly reduced hippocampal MFN2 expression, accompanied by mitochondrial-associated ER membrane (MAM) disruption and mitochondrial dysfunction. MFN2 overexpression effectively reversed sevoflurane-induced neuronal apoptosis and cognitive deficits (Zhu et al., 2024). Moreover, repeated ketamine anesthesia similarly reduced MFN2 expression in hippocampal neural stem cells, leading to impaired synaptic plasticity and long-term cognitive deficits (Huang et al., 2024a).

Dynamin-related protein 1 (Drp1) is a key regulator of mitochondrial dynamics, maintaining homeostasis by mediating mitochondrial fission (Schmitt et al., 2018; Fan et al., 2021b). Mitochondrial Drp1 levels were significantly elevated in the hippocampus of PND model mice, contributing to mitochondrial dysfunction and impaired neuronal viability (Huang et al., 2024b; Yang et al., 2022). Surgery inhibited the cold-inducible RNA-binding protein (Cirp)/thioredoxin 1 (Trx1) pathway, disrupting the cytoplasmic–mitochondrial distribution of Drp1. In HT-22 cells treated with LPS for 24 h, inflammation increased Drp1 phosphorylation at Ser616, and pharmacological inhibition of Drp1 restored autophagic function impaired by excessive mitochondrial fission (Li et al., 2023b). Recent studies have shown that surgery-induced neuroinflammation triggers mitochondrial fission in microglia, leading to cytoplasmic release of mitochondrial DNA. This release activates the cGAS–STING signaling pathway, promoting microglial activation and NLRP3 inflammasome assembly, thereby exacerbating postoperative cognitive impairment (Yang et al., 2024b).

**Fig. 6.**
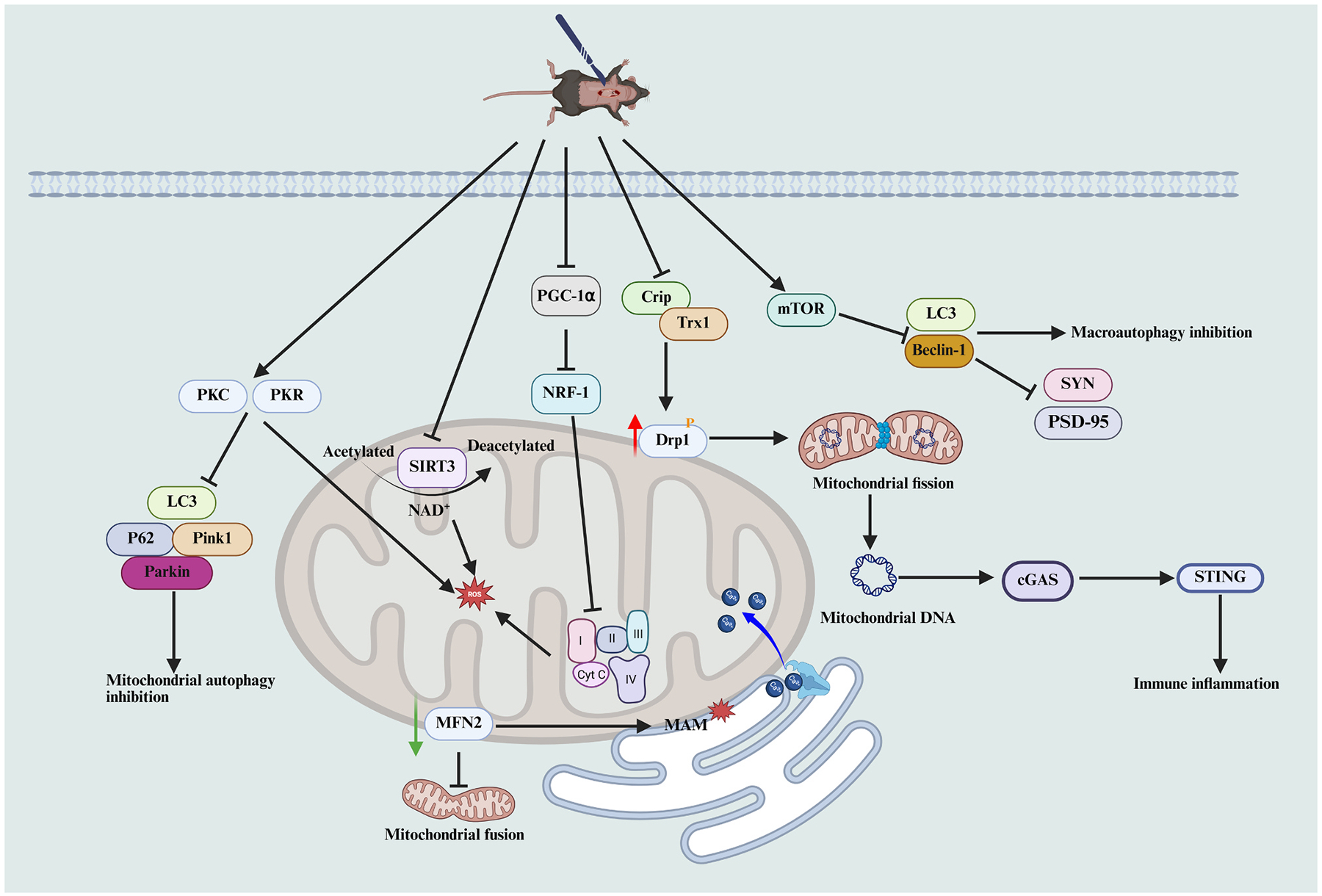
Mitochondrial and autophagy dysfunction in postoperative neurocognitive disorders. Downregulation of MFN2 disrupts mitochondrial fusion and weakens mitochondria–ER tethering at MAM. Reduced PGC-1α/NRF-1 signaling diminishes respiratory chain complex expression, leading to energy deficits and ROS accumulation, while loss of SIRT3 deacetylase activity further exacerbates oxidative stress. In parallel, surgical suppression of the CIRP/Trx1 pathway facilitates Drp1 activation, driving excessive mitochondrial fission and the release of mitochondrial DNA, which activates the cGAS–STING pathway and triggers immune inflammation. Activation of the PKC/PKR pathway inhibits mitochondrial autophagy, whereas mTOR-driven LC3/Beclin-1 suppression blocks macroautophagy, reducing SYN and PSD-95 expression, impairing synaptic plasticity, and aggravating neuronal injury. Collectively, these mitochondrial and autophagy impairments converge to amplify neuroinflammation, promote synaptic damage, and accelerate postoperative cognitive decline.

In a tibial fracture–induced PNDs mouse model, hippocampal expression of PPARγ coactivator-1α (PGC-1α) and nuclear respiratory factor 1 (NRF-1) was significantly downregulated, leading to reduced expression of mitochondrial respiratory chain complex subunits (He et al., 2022). Impaired mitochondrial respiration resulted in energy deficits, reduced membrane potential, and elevated ROS levels, thereby exacerbating postoperative cognitive dysfunction. In vitro studies confirmed that neuroinflammation is a primary driver of mitochondrial respiratory impairment in hippocampal neurons. Additionally, anesthesia/surgery-induced mitochondrial oxidative stress was linked to reduced expression of the NAD^+^-dependent deacetylase Sirtuin 3 (SIRT3) (Liu et al., 2021a). SIRT3 overexpression attenuated surgery-induced mitochondrial oxidative stress and neuroinflammation, thereby enhancing hippocampal synaptic plasticity and facilitating cognitive recovery (Fig. 6).

In summary, anesthesia- and surgery-induced PNDs are frequently associated with varying degrees of mitochondrial dysfunction. Preserving mitochondrial homeostasis appears critical for mitigating cognitive decline. However, most current evidence is derived from preclinical models, and the precise molecular events linking mitochondrial impairment to PNDs remain incompletely defined. Moreover, the relative contributions of mitochondrial dynamics, bioenergetics, and redox imbalance likely vary across surgical types, ages, and brain regions, warranting further mechanistic and translational studies to clarify therapeutic potential.

## 7. Autophagy dysfunction

Mitochondrial autophagy, closely regulated by mitochondrial dynamics, plays a key role in the pathogenesis of PNDs. Cellular autophagy is also a critical mechanism underlying postoperative cognitive impairment. Surgery-induced mitochondrial dysfunction and autophagy suppression are modulated by protein kinase C (PKC) and protein kinase RNA-activated (PKR) signaling pathways (Lu et al., 2022). Inhibition of PKC/PKR signaling alleviated autophagy-related neurocognitive deficits. Additionally, multiple studies have reported that anesthesia and surgery activate the mechanistic target of rapamycin (mTOR) pathway, which negatively regulates cellular autophagy and contributes to post-operative cognitive impairment (Gao et al., 2021b; Chen et al., 2022c; Jiang et al., 2020). mTOR pathway activation suppresses the expression of autophagy-related proteins, leading to impaired synaptic plasticity as well as increased tau hyperphosphorylation and amyloid-β (Aβ) accumulation in the hippocampus (Fig. 6).

## 8. Exploring drugs for PNDs: insight from preliminary animal research

Extensive preclinical research has investigated the therapeutic potential of various bioactive compounds for PNDs, primarily targeting key pathological mechanisms—most notably neuroinflammation. The following sections summarize these recent advances and their mechanistic underpinnings.

### 8.1. Bioactive molecules that regulate microglial activation

Targeting neuroinflammation by inhibiting microglial activation has become a central strategy in current drug development for PNDs. Bioactive compounds, including minocycline, galectin-1, TGF-β1, cannabinoid receptor 2 agonists, acetate, midbrain astrocyte-derived neurotrophic factor, esketamine, and histamine H2/H3 receptor agonists, can attenuate surgery- or anesthesia-induced proinflammatory microglial polarization (Gao et al., 2024; Shen et al., 2021; Lin et al., 2023; Sun et al., 2017; Chen et al., 2020; Wen et al., 2020, 2024).

Kv1.3 potassium channel blocker phenoxyalkoxypsoralen-1 (PAP-1) effectively suppresses microglia-mediated neuroinflammation without impairing tissue repair after fracture surgery (Lai et al., 2020). Additionally, URMC-099, a mixed lineage kinase 3 (MLK3) inhibitor, has been shown to suppress microglial activation while preserving the peripheral innate immune response essential for fracture healing (Miller-Rhodes et al., 2019). Thus, PAP-1 and URMC-099 represent promising therapeutic candidates for preventing cognitive impairment associated with fracture-related surgeries. However, further studies are needed to comprehensively assess their effects on surgical outcomes and validate their safety and efficacy before clinical translation.

Plant-derived natural products, including traditional Chinese herbs, have shown considerable neuroprotective potential. The active extract of *Sigesbeckia orientalis* L. attenuated hippocampal microglial activation, suppressed proinflammatory cytokine release, and inhibited activation of the JNK and NF-κB signaling pathways (Chu et al., 2022). Natural plant-derived anthocyanidins (ANTs) alleviated microglial activation and laparotomy-induced postoperative cognitive impairment by inhibiting MLK3 and its downstream signaling cascades (Zhang et al., 2020b). Researchers administered β-caryophyllene (BCP) and ellagic acid (EA) to PND model mice, demonstrating that these plant-derived compounds attenuate microglial proinflammatory polarization and improve post-operative cognitive function via activation of the CB2 receptor (CB2R) and insulin-like growth factor 1 (IGF-1) signaling pathways, respectively (Chen et al., 2023c, 2024b). Another study demonstrated that pre- and postoperative administration of two ginseng-based traditional Chinese medicines effectively reduced neuroinflammation and cognitive impairment in aged rats following surgery (Zhang et al., 2018b). The therapeutic benefits of ginseng were further attributed to its modulation of mitochondrial energy metabolism and oxidative stress, primarily mediated by its active component, Ginsenoside Rg1 (Miao et al., 2019). In summary, these findings highlight the therapeutic potential of plant-based natural products and traditional Chinese medicines in attenuating postoperative neuroinflammation and cognitive dysfunction.

Given the pivotal role of astrocyte activation in promoting microglial-driven neuroinflammation after surgery, targeting astrocytes may offer a promising therapeutic strategy for PNDs. Maresin 1, an anti-inflammatory lipid mediator derived from docosahexaenoic acid and produced by macrophages during inflammation resolution, has been shown to reduce hippocampal neuroinflammation by inhibiting astrocyte activation via modulation of the NF-κB signaling pathway (Li et al., 2023c; Yang et al., 2019). Gap junction protein Connexin43 (Cx43) is a critical component of the astrocytic network. Anesthesia- and surgery-induced uncoupling of GJs-Cx43 contributes significantly to CNS dysfunction. Intraperitoneal administration of the Cx43 enhancer Danegaptide promotes astrocytic network repair and alleviates anesthesia-induced cognitive impairment (Dong et al., 2022a, 2022b). Additionally, pharmacological inhibition of A1-specific astrocyte activation presents a promising therapeutic strategy for PNDs. However, further studies are needed to validate its efficacy and translational potential.

### 8.2. Bioactive molecules targeting NLRP3

NLRP3-mediated inflammasome activation and pyroptosis following surgery highlight NLRP3 as a key therapeutic target. Peritoneal administration of the NLRP3 inhibitor MCC950 attenuated hippocampal inflammasome activation and significantly reduced the expression of proinflammatory cytokines in surgical mice (Fu et al., 2020). A biomimetic peptide derived from the pro-resolving molecule Annexin-A1 was shown to inhibit NLRP3 inflammasome activation and hippocampal microglial activation, thereby improving postoperative memory deficits (Zhang et al., 2022b). Dexmedetomidine (DEX), a highly selective α2-adrenergic receptor (α2-AR) agonist, has been extensively studied for its neuroprotective effects. Pretreatment with DEX has been shown to inhibit NLRP3 inflammasome activation through multiple mechanisms, including reducing monocyte/macrophage infiltration, suppressing HMGB1/NF-κB signaling and P2X4R channel activity, modulating miRNA expression, and promoting NLRP3 degradation via the autophagy–ubiquitin pathway (Wang et al., 2022c; Kii et al., 2022; Li et al., 2024b; Deng et al., 2024b; Zhang et al., 2021). In addition, the cholesterol-lowering agent atorvastatin reduces hippocampal neuronal apoptosis and blood–brain barrier disruption in surgical mice by inhibiting NLRP3 inflammasome activation (Liu et al., 2021b). These findings highlight the potential of targeting NLRP3 inflammasome activation to attenuate PNDs.

### 8.3. Bioactive molecules targeting tau

Excessive tau acetylation, phosphorylation, and ectopic expression in the nervous system after surgery are key contributors to postoperative cognitive decline. In PNDs model mice, hippocampal expression of α7nAChR is significantly reduced. Varenicline, a selective α7nAChR agonist, has been shown to alleviate postoperative cognitive impairment by mitigating DNA damage and correcting tau mislocalization (Huang et al., 2018).

One study demonstrated that 6-day-old male mice exhibited greater sensitivity to sevoflurane-induced tau phosphorylation compared to 60-day-old males, suggesting age-dependent vulnerability to anesthesia-induced tau pathology (Yang et al., 2021). The heightened sensitivity in neonatal male mice is largely attributed to low endogenous testosterone levels. Testosterone administration alleviated sevoflurane-induced cognitive impairment by suppressing tau and GSK-3β phosphorylation and their interaction. Similarly, pretreatment with the natural plant compound gastrodin inhibited phosphorylation of tau and GSK-3β, thereby reducing neuroinflammation and improving cognitive outcomes in mice undergoing laparotomy (Wang et al., 2021). These findings suggest that targeting post-translational modifications of pathogenic proteins offers a promising therapeutic strategy for PNDs and underscore the potential of epigenetic modification–based drug development.

### 8.4. Bioactive molecules that regulate mitochondrial and synaptic function

Surgery-induced mitochondrial dysfunction and synaptic injury are key contributors to postoperative cognitive impairment. The mitochondrial energy enhancer coenzyme Q10 has been shown to restore synaptic protein expression and improve mitochondrial energy supply following anesthesia, thereby alleviating memory deficits (Xu et al., 2017b). WS635, a newly developed compound, effectively attenuates postoperative synaptic damage and mitochondrial dysfunction in the hippocampus and cortex of mice (Lin et al., 2021b). Notably, although WS635 is a cyclosporin A (CsA) analogue, it lacks immunosuppressive activity, broadening its therapeutic potential in PNDs. Additionally, elamipretide, a synthetic mitochondrial-targeted antioxidant tetrapeptide, has been shown to restore mitochondrial and synaptic function while also inhibiting NLRP3 inflammasome–mediated pyroptosis (Zuo et al., 2020). Clemastine, an antihistamine, has emerged as a potential therapeutic agent for PNDs. Drug repurposing studies have demonstrated its ability to promote remyelination and restore synaptic function (Mei et al., 2014; Wang et al., 2018b). A recent study demonstrated that clemastine enhances neuronal myelin regeneration and ameliorates synaptic deficits in mice subjected to isoflurane anesthesia and laparotomy (Wu et al., 2021). Given that clemastine is an FDA-approved drug, it may represent a safe and reliable option for PNDs.

Collectively, therapeutic strategies targeting mitochondrial and synaptic dysfunction show great promise for the treatment of PNDs. The development of non-immunosuppressive compounds and mitochondrial-targeted antioxidants offers new avenues for intervention with broader applicability and reduced adverse effects.

### 8.5. Bioactive molecules that regulate the intestinal microenvironment

Perioperative gut dysbiosis is a key contributor to impaired neurocognitive recovery. In a mouse model of tibial fracture fixation, cefazolin administered pre- and postoperatively alleviated BBB disruption and cognitive deficits. Although surgery alone did not significantly alter gut microbiota, it induced shifts in bacterial composition (elevated *Bacteroidetes*, *γ/δ-* and *ε-Proteobacteria*, and reduced *β-Proteobacteria*) which were reversed by cefazolin (Luo et al., 2021b). Moreover, while cefazolin reverses surgery-induced reductions in SCFAs, its administration in healthy mice also decreases SCFAs levels. Early studies revealed that cefazolin induces gut microbiota dysbiosis in both healthy and surgical mice, with dysbiosis persisting for at least 19 days postoperatively in surgical models. This prolonged imbalance may underlie the colonic inflammation observed following cefazolin treatment in both contexts (Liang et al., 2018). These findings indicate that although cefazolin may ameliorate postoperative cognitive deficits, it also poses a risk of inducing gut microbiota dysbiosis and sustained inflammation. Therefore, its potential adverse effects warrant careful evaluation in perioperative applications.

A recent study investigated the therapeutic potential of itaconate, an immunomodulatory metabolite that regulates inflammation and cellular metabolism, in a laparotomy-induced PNDs mouse model. Itaconate attenuated surgery-induced neuroinflammation and gut microbiota disturbances, while promoting endogenous neurogenesis via activation of Nrf2 signaling (Kong et al., 2024). However, its effects in healthy mice remain unclear due to the lack of an itaconate-treated healthy control group, highlighting the need for further evaluation of its safety profile.

## 9. Concluding remarks

PNDs are multifactorial syndromes involving a complex interplay between neuroinflammation, neurotransmitter disturbances, epigenetic modifications, and gut–brain axis dysregulation. Current evidence emphasizes that microglial activation and NLRP3 inflammasome-mediated pyroptosis play central roles in the initiation and progression of neuroinflammation. Anesthesia and surgical stress disrupt key neurotransmitter systems, such as GABA_A_ and NMDA receptors, impairing synaptic plasticity and cognitive function. In parallel, epigenetic mechanisms contribute to long-term transcriptional and structural alterations in the brain.

Emerging studies also highlight the role of gut microbiota dysbiosis in modulating neuroinflammation and neural function, suggesting that the gut–brain axis represents a promising therapeutic target. Furthermore, the preclinical success of various bioactive compounds—including anti-inflammatory agents, mitochondrial protectants, and traditional Chinese medicines—underscores the potential for multi-modal interventions in PND prevention and treatment.

Nevertheless, the field faces critical challenges. Most current studies rely on rodent models, and the translation of findings into clinical practice remains limited. There is a pressing need to strengthen high-quality clinical trials and develop unified diagnostic criteria, objective biomarkers, and precise risk stratification tools. Future research should also explore individualized therapeutic strategies based on patient-specific neuroimmune and metabolic profiles.

In summary, unraveling the intricate pathophysiology of PNDs and identifying targeted, mechanism-based interventions will be essential for reducing their incidence and improving cognitive outcomes in surgical patients, especially the elderly.

## Figures and Tables

**Table1:** Mechanisms of ncRNA involvement in PNDs.

PNDs models	NcRNA	Changes in brain	Target Gene/signal pathway	Regulatory effects	Refers
Open fracture tibia	miRNA–181b–5p	Decreased	TNF-α	Aggravate TNF-α-induced neuroinflammation	152
Cardiopulmonary bypass	miRNA–124–3p	Decreased	LPIN1	Increase neuroinflammation and cell apoptosis	153
Cardiopulmonary bypass	miRNA–214–3p	Decreased	PTGS2	Induce inflammation and oxidative stress	154
Unilateral nephrectomy	miRNA-let–7b	Increased	TLR7	Aggravate TLR7-induced neuroinflammation	156
Open fracture tibia	miRNA–146a	Increased	IRAK1, TRAF6	Relieve neuroinflammation	157
Isoflurane anesthesia	miR–106a	Increased	LIMK1	Enhance apoptosis signal	159
Sevoflurane anesthesia	miR–125b–5p	Increased			160
Sevoflurane anesthesia	lncRNA-Rian	Decreased	miRNA–143–3p/LIMK1	Enhance cell damage and apoptosis	163
Osteotomy	lnc-MEG3	Decreased	has-miR–106a–5p/SIRT3	Enhance inflammation and oxidative stress	164
Hepatic ischemic reperfusion	lnc-NET1	Increased	miRNA–122–5p/Wnt1/β-catenin	Reduce cell activity and promote cell apoptosis	165
Intramedullary fixation of tibial fractures	circRNA-AKT3	Decreased	miR–106a–5p/HDAC4/MEF2C	Promote apoptosis of hippocampal neurons	167

## Data Availability

No data was used for the research described in the article.
